# A novel bispecific c-MET/PD-1 antibody with therapeutic potential in solid cancer

**DOI:** 10.18632/oncotarget.16173

**Published:** 2017-03-14

**Authors:** Zu-Jun Sun, Yi Wu, Wei-Hua Hou, Yu-Xiong Wang, Qing-Yun Yuan, Hui-Jie Wang, Min Yu

**Affiliations:** ^1^ Key Laboratory of Metabolism and Molecular Medicine, Ministry of Education and Department of Biochemistry and Molecular Biology, School of Basic Medical Science, Fudan University, Shanghai, China; ^2^ Department of Medical Oncology, Shanghai Cancer Center and Department of Oncology, Shanghai Medical College, Fudan University, Shanghai, China

**Keywords:** cellular-mesenchymal to epithelial transition factor, programmed death-1

## Abstract

The bispecific antibody is a novel antibody, which can target two different antigens and mediate specific killing effects by selectively redirecting effector cells to the target cells. Here, we designed and synthesized a bispecific antibody (BsAb) that can bind cellular-mesenchymal to epithelial transition factor (c-MET, overexpressed in several human solid tumor), and programmed death-1 (PD-1, involved in cancer cell immune evasion) with high affinity and specificity. We found that BsAb can induce the degradation of c-MET protein in cancer cells, including MKN45, a gastric cancer cell line, and A549, a lung cancer cell line. BsAb inhibited hepatocyte growth factor (HGF)-mediated proliferation, migration, and antiapoptosis, and downregulated HGF-stimulated phosphorylation of c-MET, protein kinase B (AKT), and extracellular signal-regulated kinase (ERK1/2). BsAb can also rescue T cell activation. Furthermore, xenograft analysis revealed that BsAb markedly inhibits the growth of subcutaneously implanted tumors and chronic inflammation. On the basis of these results, we have identified a potential bispecific drug, which can effectively target c-MET and PD-1 for the treatment of human solid cancers.

## INTRODUCTION

Cellular-mesenchymal to epithelial transition factor (c-MET) is confirmed as the only high affinity receptor that binds hepatocyte growth factor (HGF) [1], which mediates cell morphogenesis, mitogenesis, angiogenesis, and cytoprotection *in vitro* [2, 3]. c-MET is overexpressed in a broad spectrum of human solid tumors [2, 4], and once activated, promotes tumor progression, invasion, metastasis, and angiogenesis [5]. c-MET is also overexpressed in human glioblastomas, and expression levels correlate with glioma malignancy grade and vascularity, promoting glioma growth and angiogenesis *in vivo* [5–10]. Activation of the HGF/c-MET pathway in various solid tumors can stimulate lymphangiogenesis, leading to lymph node metastasis [11]. Consequently, c-MET has become a leading target candidate for cancer therapy. Currently, commercial c-MET inhibitors used in second-line treatment in phase 2 clinical trials significantly prolong progression time and survival of patients with hepatocellular carcinoma [12, 13]. However, several *in vivo* studies published showed that some c-MET inhibitors carry potential side effects, such as heart rate acceleration, cardiac muscle denaturation, renal toxicity, and body weight reduction [14–16]. Following clinical trials, monoclonal antibodies against growth factors or their receptors have been approved for cancer therapy. Nevertheless, targeting c-MET with monoclonal antibodies has proved difficult because most antibodies have intrinsic agonistic activity [17, 18].

Programmed death-1 (PD-1) is an immunoglobulin superfamily member expressed on activated and exhausted T cells, which can also recruit regulatory T (Treg) cells [19]. Programmed death-ligand 1 (PD-L1), the primary ligand for PD-1, is broadly expressed by most cell types, including dendritic cells (DCs), as well as by tumor cells [20–22]. Upon ligation, the PD-1/PD-L1 pathway recruits Src homology 2 domain-containing phosphatase-2 (SHP-2) to control peripheral tolerance [19, 23]. PD-L1 is upregulated in the tumor microenvironment in response to inflammatory stimuli, and the PD-1/PD-L1 pathway can inhibit T cell-mediated anti-tumor responses [23, 24]. Monoclonal antibodies blocking coinhibitory immune checkpoint receptors (e.g., PD-1/PD-L1) show remarkable efficacy against many cancers. For example, anti-PD-1 antibody produced objective clinical responses in approximately 20-25% of patients with non-small-cell lung cancer (NSCLC), melanoma, and renal-cell cancer [25, 26], and anti-PD-1/PD-L1 showed objective responses in NSCLC as a monotherapy, with evidence for markedly increased overall survival in second-line treatment reported in patients with adenocarcinoma and squamous cell carcinoma [27–30]. Recently, the FDA approved two agents blocking PD-1 (nivolumab and pembrolizumab) for the treatment of metastatic melanoma [31, 32]. Ipilimumab, a monoclonal antibody that works to activate the immune system by targeting CTLA-4, combined with nivolumab attained intense and synergistic therapeutic effects in the treatment of a deadly form of skin cancer [33–34]. Ipilimumab combined with chemotherapy showed a modest degree of clinical activity in the treatment of patients with metastatic NSCLC [35]. However, it has to be noted that systemic administration of PD-1/PD-L1 blocking antibodies carries potential side effects, such as persistent high fever and breakdown of peripheral tolerance [36].

In the present study, a novel targeted c-MET and PD-1 BsAb was developed in our laboratory, that can bind human c-MET and PD-1 with high affinity and specificity, and induce the degradation of c-MET in multiple cancer cell types, including MKN45, a gastric cancer cell line, and A549, a lung cancer cell line. Our BsAb can inhibit HGF-induced growth and migration of c-MET-addicted tumor cells, promote the apoptosis of tumor cells, and rescue IL-2 secretion of Jurkat T cells. BsAb can also inhibit HGF-stimulated c-MET autophosphorylation of Tyr1234/1235 in the activation loop, which activates downstream molecules, such as protein kinase B (AKT) and extracellular signal-regulated kinase (ERK). We have further identified that our BsAb could potently inhibit tumor growth and inflammatory factor secretion *in vivo*, implicating its immunotherapeutic potential in human solid tumor treatment.

## RESULTS

### Expression of c-MET and PD-L1 by tumor cells

**Figure 1:**
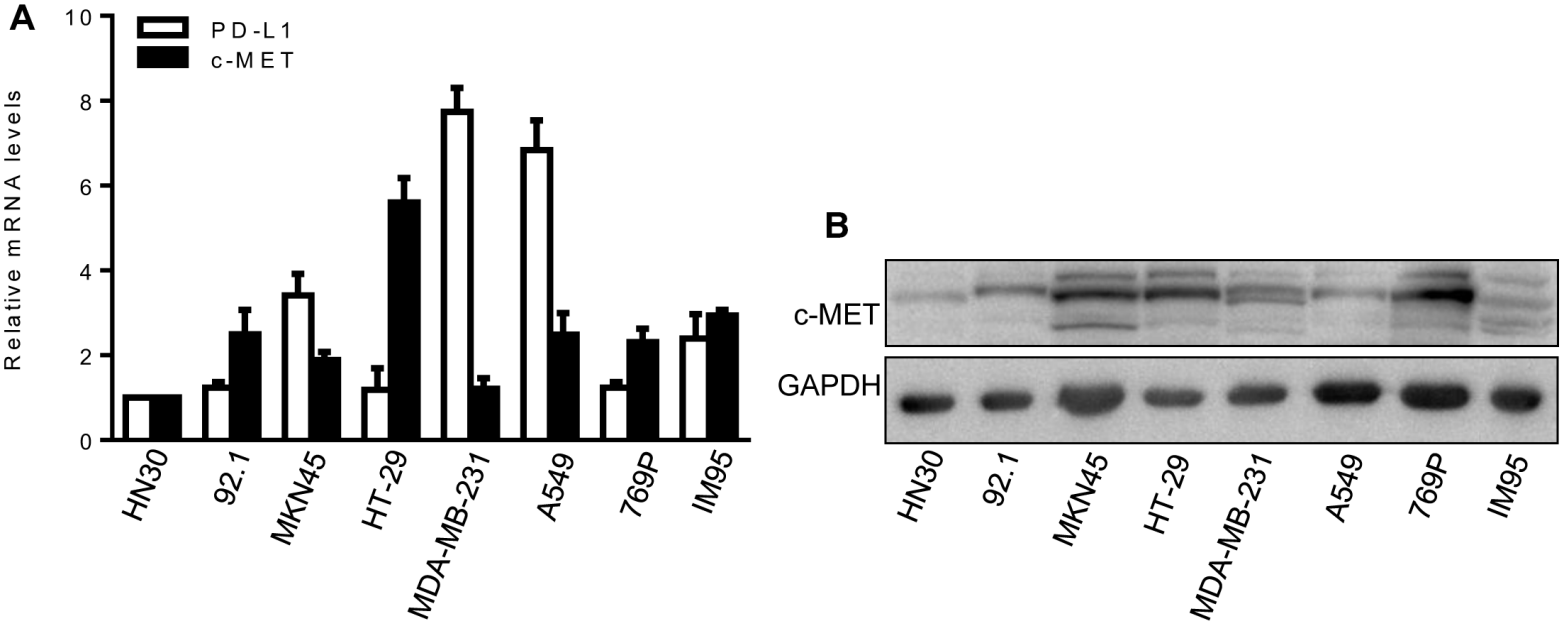
Expression of c-MET and PD-L1 in cancer cell lines **(A)** c-MET and PD-L1 mRNA expression were detected by qPCR. HN30 cells as the calibrator sample. Each experiment was repeated three times. **(B)** c-MET protein expression was detected by western blot analysis. GAPDH expression was used as an internal control.

First, we evaluated c-MET and PD-L1 mRNA expression in eight human cancer cell lines using real-time quantitative polymerase chain reaction (qPCR). All eight cell lines expressed c-MET at a relative high levels, but only four (MKN45, MDA-MB-231, A549, and IM95)expressed PD-L1 at a relative high levels (Figure 1A). Next, we detected c-MET protein expression by western blot. The results showed that all eight cancer cell lines also expressed c-MET protein at a relative high levels (Figure 1B). Finally, we determined whether the above eight cancer cell lines can produce xenografts in NOD/SCID mice. Three of the eight cell lines produced xenografts well (MKN45, A549, and IM95) (data not shown), hence, MKN45 and A549 cell lines were selected for use in this study, both of which express relative high levels of c-MET and PD-L1, and effectively produce xenografts.

### BsAb inhibits HGF-stimulated cancer cell proliferation, migration, and antiapoptosis

**Figure 2:**
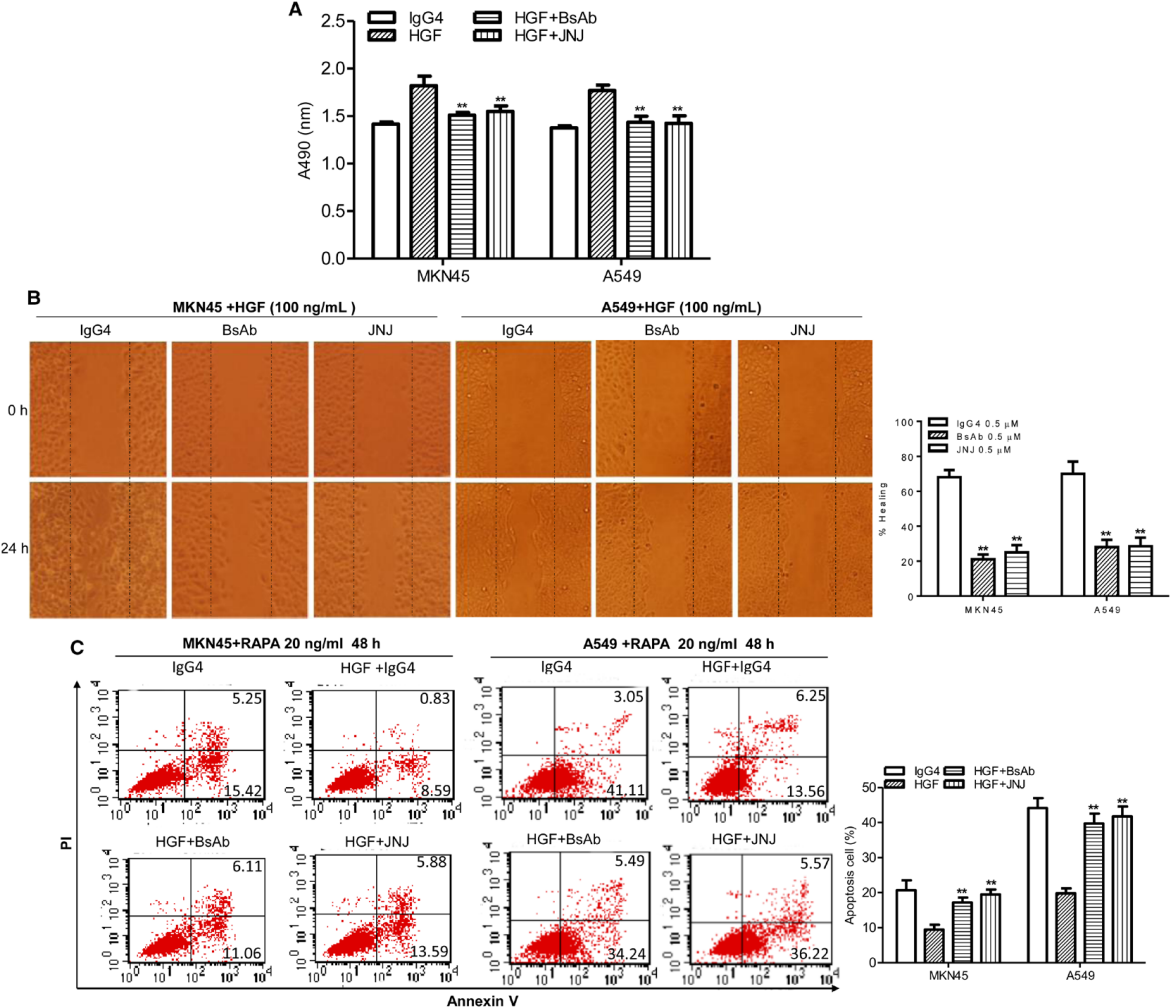
BsAb inhibits HGF-induced cancer cells proliferation, migration, and apoptosis resistance **(A)** The viability of MKN45 and A549 cells were assessed by MTS assay after treatment with HGF (100 ng/mL), IgG4 (0.5 μM), BsAb (0.5 μM), and JNJ (0.5 μM) for 72 h. Each experiment was repeated 3 times. **: *P* < 0.01. **(B)** Wound healing assay. Cancer cells were cultured to confluency on plastic dishes. Next day a linear scrape wound was made using a sterile tip, and cells were treated as described in the materials and methods section. (Original magnification, 100×). Each experiment was repeated 3 times. **: *P* < 0.01. **(C)** Cancer cells were incubated with BsAb (0.5 μM) for 8 h or JNJ (0.5 μM) for 2 h and then treated with combinations of HGF (100 ng/mL) and RAPA. After 48 h treatment, apoptotic cells stained with annexin V and propidium iodide, and analyzed by flow cytometry. Each experiment was repeated 3 times and the results were shown mean ± SD. **: *P* < 0.01.

Our BsAb has two targets, and can block c-MET and PD-1. To test the effect of BsAb targeting of c-MET on tumor cells growth, tumor cells were treated with or without BsAb for 8 h and JNJ 38877605 (JNJ, a known c-MET inhibitor) for 2 h before HGF treatment. Cell viability was then measured using the MTS assay. BsAb significantly inhibited cancer cell growth, and had the same effect as JNJ (Figure 2A). In migration assays, BsAb significantly inhibited MKN45 and A549 cells migration (Figure 2B). The same effect was observed in JNJ treated group (Figure 2B).

c-MET rescued apoptosis in renal cancer cells [37], and inhibition of c-MET signaling increased mitochondrial release of cytochrome C and the Bax/Bcl2 ratio [38]. We therefore evaluated the effect of BsAb on c-MET-mediated signaling in the regulation of cancer cell death. Tumor cells were treated with BsAb for 8 h or JNJ for 2 h, and then treated with combinations of HGF and rapamycin (RAPA) for 48 h. Cells were stained with annexin V and propidium iodide (PI), and analyzed by flow cytometry to measure the apoptotic index. HGF significantly rescued RAPA-induced cellular apoptosis (Figure 2C, left), with a reduction from 20.67% to 9.43% in MKN45 cells; nevertheless, our BsAb markedly inhibited this effect. With BsAb pre-treatment, the percentage of RAPA-induced apoptotic MKN45 cells increased from 9.43% to 17.17%. JNJ produced the same effect as BsAb, and the same phenomenon was shown in A549 cells (Figure 2C, right). Altogether, our results indicate that BsAb inhibits HGF-mediated proliferation, migration, and antiapoptosis effects in cancer cells, and could serve as an inhibitory c-MET antibody.

### BsAb inhibits HGF-triggered c-MET downstream molecules and promotes c-MET degradation

**Figure 3:**
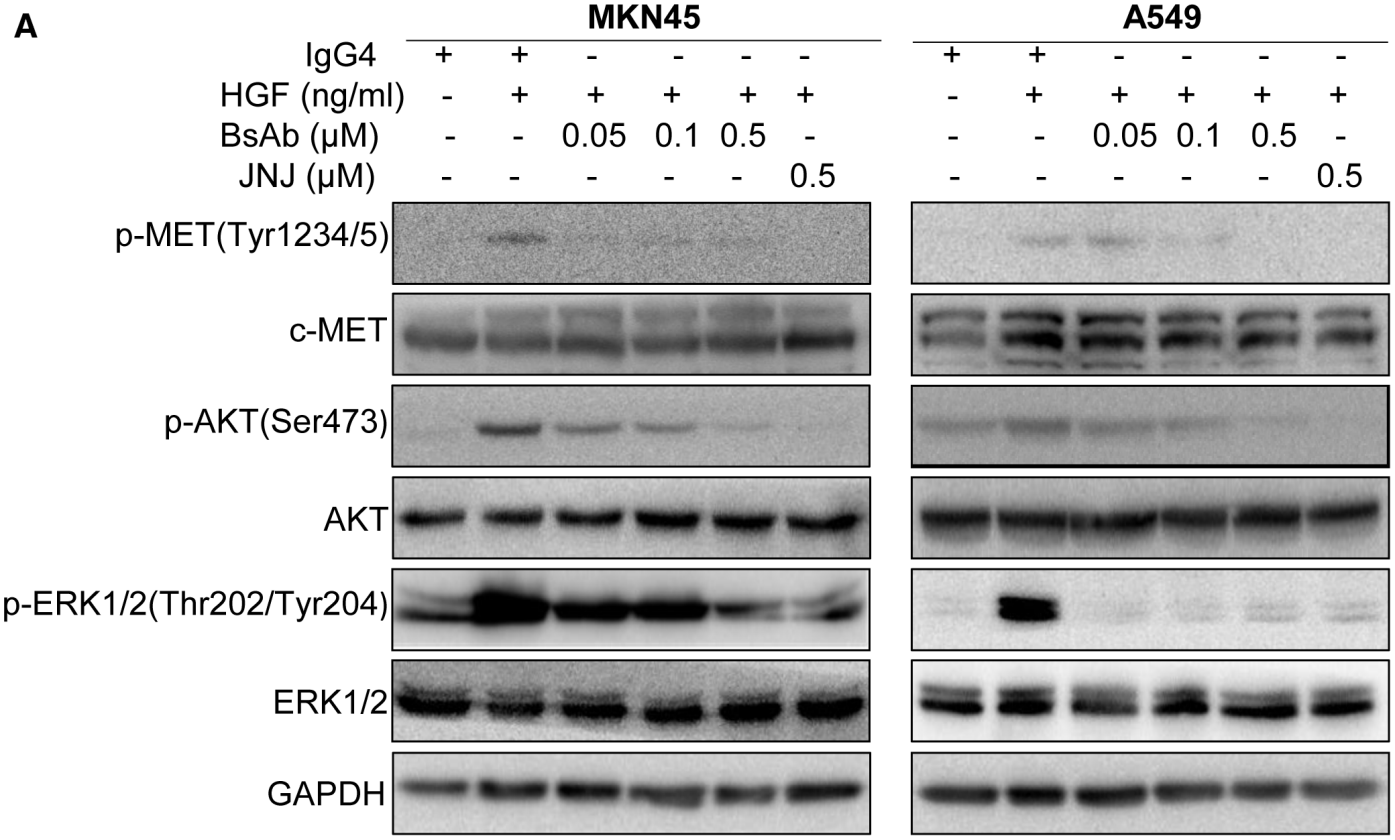
BsAb inhibits HGF-induced c-MET and its downstream molecules **(A)** MKN45 and A549 cells were treated with the indicated concentrations of BsAb for 8 h or JNJ for 2 h, and then stimulated with or without HGF (100 ng/mL) for 30 min. Total cell lysates were evaluated by western blot using specific antibodies. GAPDH expression was used as an internal control.

The HGF/c-MET signal pathway plays a significant role in tumor development and metastasis [39], often through activation of downstream molecules, including AKT and ERK1/2 pathways. In the present study, we found that HGF-mediated upregulation of p-c-MET in MKN45 and A549 cells was completely inhibited with BsAb treatment, even more profoundly efficiency than JNJ treatment (Figure 3). Upregulation of p-AKT was observed in response to HGF stimulation that was also inhibited by BsAb and JNJ. Furthermore, upregulation of p-ERK1/2 was also inhibited by BsAb and JNJ treatment.

**Figure 4:**
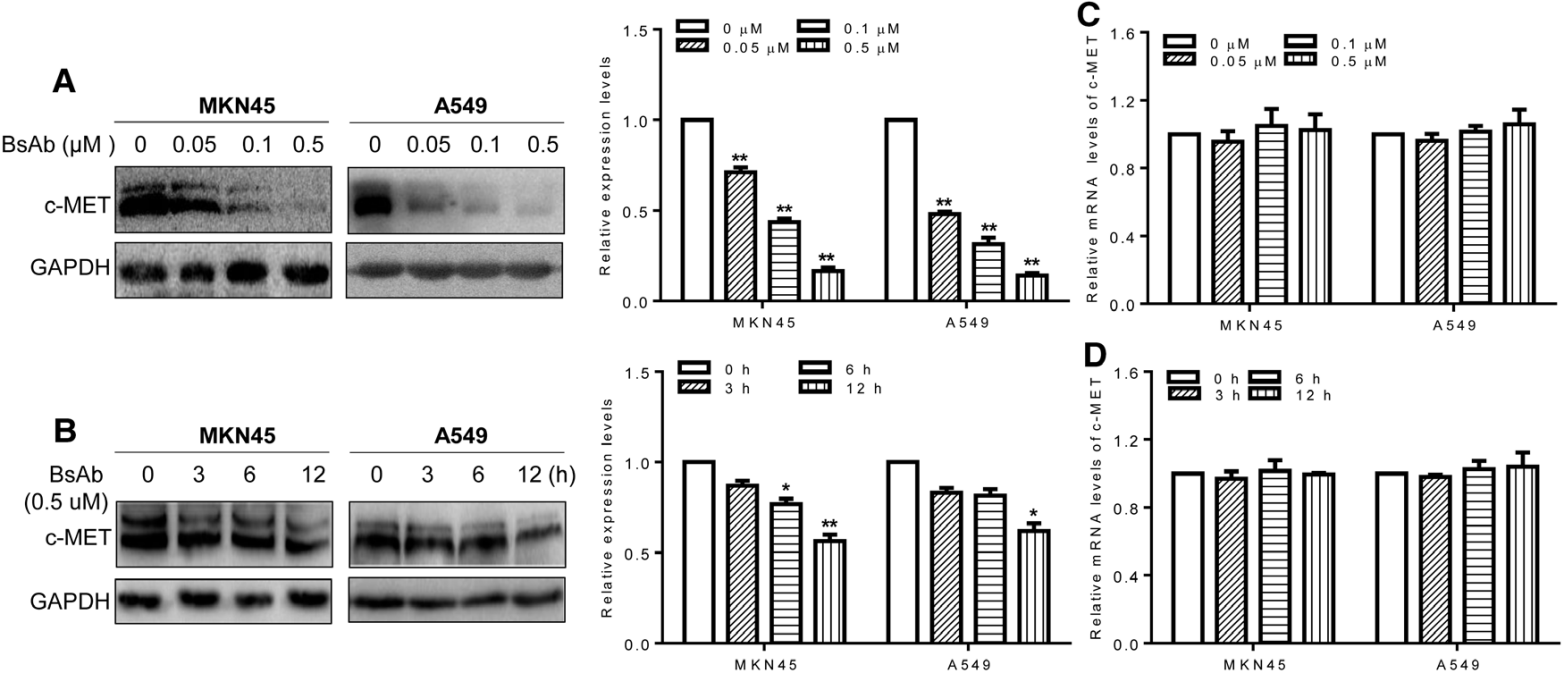
BsAb promotes c-MET protein degradation **(A, B)** Cells were treated with the indicated concentrations of BsAb for 24 h **(A)** or for the indicated time **(B)**. Total cell lysates were evaluated by western blot using anti-c-MET antibodies. Relative c-MET mRNA expression was evaluated by qPCR. **(C, D)** Cells were treated with the indicated concentrations of BsAb for 24 h **(C)** or for the indicated time **(D)**. BsAb (0 μM or 0 h) treatment as the calibrator sample. Above assays were repeated three times and the results were shown as mean ± SD. *: *P* < 0.05;**: *P* < 0.01

Quercetin and anti-c-MET antibodies can inhibit HGF/c-MET signaling by promoting c-MET protein degradation [40]. To clarify whether BsAb could induce c-MET degradation, western blot analysis was performed to assess c-MET stability. BsAb strongly downregulated c-MET protein expression in both dose- and time-dependent manners in the two cancer cell lines (Figure 4A, 4B). However, there was no effect on c-MET mRNA expression, assessed by qPCR (Figure 4C, 4D). These results suggest that the AKT and ERK1/2 pathways play an essential role in cancer cell proliferation, migration, and apoptosis, and BsAb could prove to be an effective inhibitor of cancer development, via c-MET targeting.

### BsAb promotes IL-2 production by Jurkat T cells

**Figure 5:**
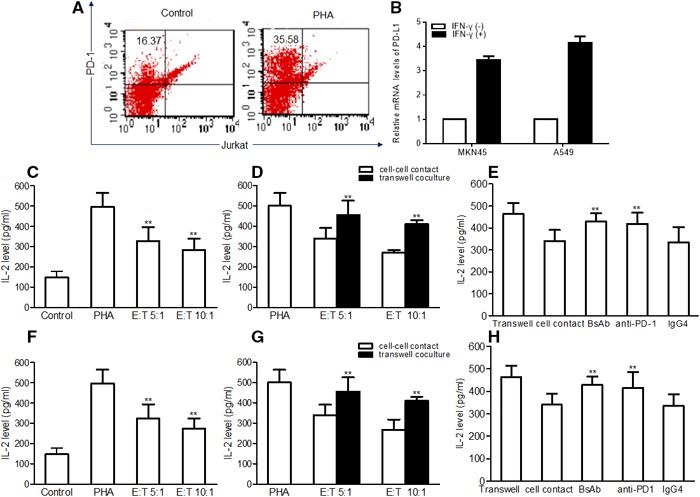
PD-L1 inhibits IL-2 production by human Jurkat T cells MKN45 and A549 cells were pretreated with IFN-γ (500 U/mL) for 48 h, washed and cocultured with human Jurkat T cells, either with cell contact or in transwell coculture, in the presence of PHA (1 μg/mL) for 48 h. IL-2 production was detected by ELISA. **(A)** The expression of PD-1 in Jurkat T cells with or without PHA stimulation by flow cytometry. **(B)** The relative mRNA expression of PD-L1 in cancer cell lines, with or without IFN-γ stimulation, detected by qPCR. IFN-γ (−) as the calibrator sample. **(C, F)** Inhibition of IL-2 production by Jurkat T cells in coculture with MKN45 **(C)** and A549 cells **(F)** at different E:T proportions. **(D, G)** Enhanced IL-2 production in transwell cocultures in MKN45 cells **(D)** and A549 cells **(G)**. **(E, H)** Restoration of IL-2 production by addition of BsAb to Jurkat T cell-contact cocultures with MKN45 cells **(E)** and A549 cells **(H)**. The assays were repeated three times and each sample has 3 holes, the results were shown as mean ± SD. **: *P* < 0.01.

Cytokine secretion is an important criterion for evaluating T cell function. To assess the effect of MKN45 and A549 cell expression of PD-L1 on T cell function, Jurkat T cells were cocultured with IFN-γ-pretreated MKN45 and A549 cells at different E:T proportions in the presence of PHA for 48 h, and then tested for IL-2 production by ELISA. Jurkat T cells were shown to express PD-1 at low levels, however, approximately 36% of Jurkat T cells expressed PD-1 after stimulation with PHA (1 μg/mL) for 48 h (Figure 5A). The relative mRNA expression of PD-L1 upregulated with IFN-γ stimulation for 48 h (Figure 5B).

MKN45 and A549 cells cocultured with Jurkat T cells at an E:T of 5:1 significantly reduced Jurkat T cell IL-2 production (Figure 5C, 5F). In a co-culture system, MKN45 and A549-associated inhibition of IL-2 secretion was shown to be cell-contact-dependent; IL-2 levels were significantly inhibited in Jurkat T cells cocultured in contact with MKN45 and A549 cells (*P*<0.01), compared to those in separated coculture. At the same time, the transwell coculture of cancer cells and Jurkat T cells also inhibited the secretion of IL-2 at lower levels (Figure 5D, 5G).

In order to verify that the cell-contact-dependent inhibition of IL-2 secretion was due to PD-L1:PD-1 interaction, BsAb or IgG4 controls were added to the coculture systems. BsAb restored the secretion of IL-2 by Jurkat T cells by more than 40%. However, addition of IgG4 control had no effect on IL-2 secretion by Jurkat T cells (Figure 5E, 5H).

From above results, we concluded that our BsAb can restore cytokine secretion by activated T cells, via interaction with PD-1, which may occur by blocking the PD-L1:PD-1 pathway.

### BsAb inhibits tumor development and inflammatory cytokine secretion *in vivo*

**Figure 6:**
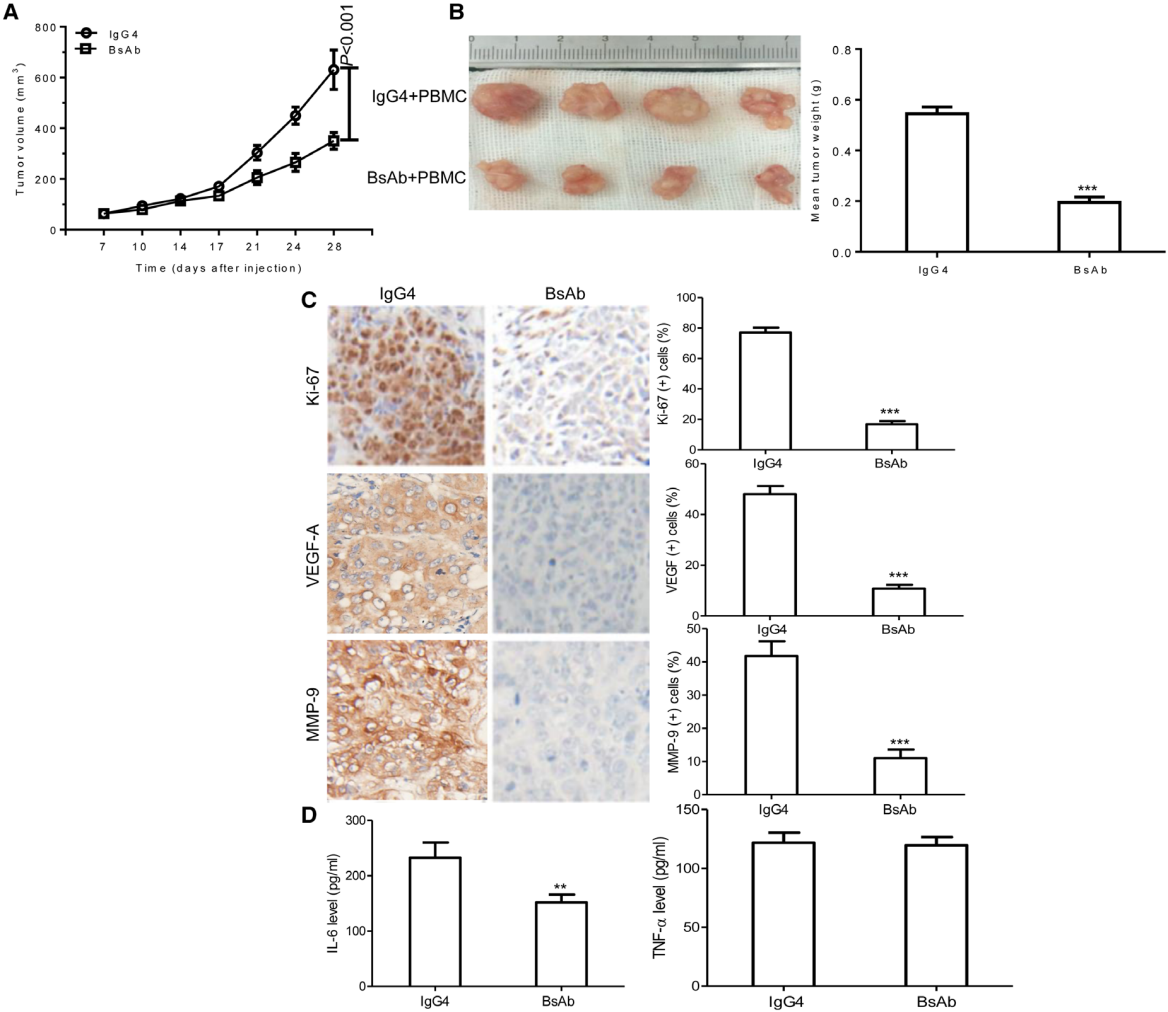
BsAb inhibits tumor development and chronic inflammation *in vivo* **(A)** Xenograft studies were performed using 5-6 week old male NOD/SCID mice (n=4/per group), by subcutaneous injection of MKN45 cells (5 × 10^6^ cells). Tumor volumes measured on indicated days were plotted for the BsAb treatment and control groups. **(B)** MKN45 tumor xenografts were stripped 4 days after the last treatment. **(C)** Semi-quantification of IHC was expressed as percentage of positively stained cells. Representative images of tumors from 5 fields randomly were counted (n=4/per group), showing Ki-67, VEGF-A, and MMP-9 (magnification: 400×). **(D)** Serum levels of IL-6 and TNF-α were measured by ELISA. The assays were repeated 2 times and each sample has 3 holes, data represent the mean ± SD of 4 individual mice per group. **: *P* < 0.01; ***: *P*< 0.001.

Finally, we evaluated the effect of BsAb on tumor development by heterotopic xenograft analysis *in vivo*. NOD/SCID mice were subcutaneously injected with MKN45 cells. After 7 days, BsAb and IgG4 were administered twice a week for 3 weeks. PBMC were injected the first and fourth time drugs were administered through the tail vein. BsAb significantly inhibited MKN45-induced tumor growth compared to IgG4, reducing the tumor volume by nearly 60% (Figure 6A), and inhibited tumor weight by approximately 50% (Figure 6B). The body weight change of mice was not significant difference (data not shown). Moreover, immunohistochemical analysis showed that BsAb significantly reduced Ki-67, VEGF-A, and MMP-9 staining (Figure 6C), which indicates it may inhibit cell proliferation, angiogenesis, and metastasis *in vivo*. In addition, we found that BsAb reduced the secretion of inflammatory cytokines *in vivo* (including IL-6, but not TNF-α) compared with control mice (Figure 6D), which suggests that BsAb treatment can inhibit chronic inflammation *in vivo*.

### BsAb inhibits tumor development beyond PD-1 antibody blockade *in vivo*

**Figure 7:**
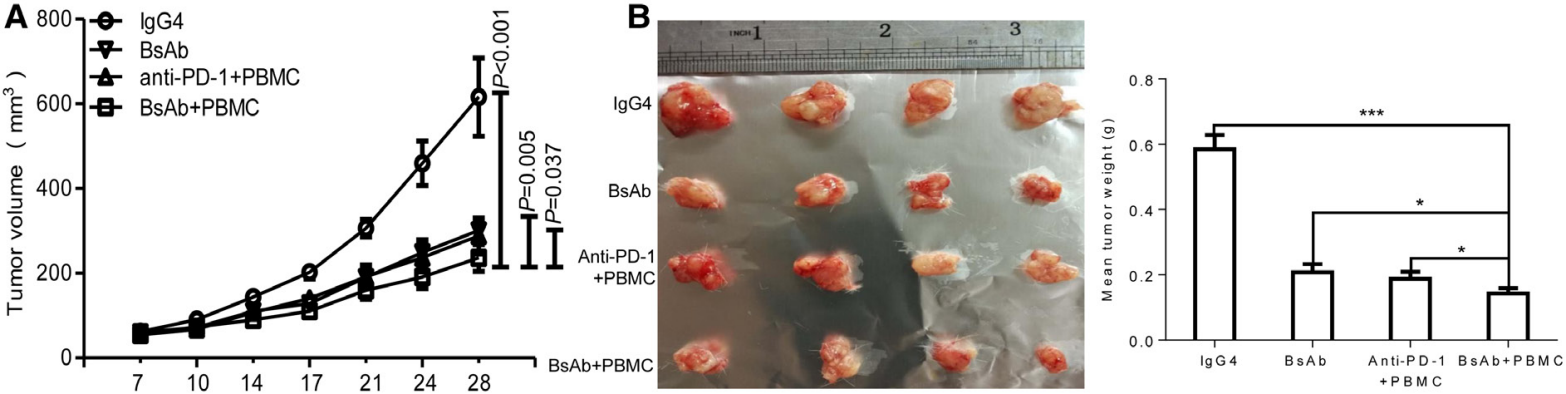
The therapeutic effect of BsAb exceeds that of PD-1 antibody in xenograft mice **(A)** Xenograft studies were performed using 5-6 week old male NOD/SCID mice (n=4/per group) by subcutaneous injection of MKN45 cells (5 × 10^6^ cells). Tumor volumes, measured on indicated days, were plotted for each treatment group and the control group. **(B)** Tumor xenografts from MKN45 were stripped 4 days after the last treatment. The assays were repeated 2 times and data represent the mean ± SD of 4 individual mice per group. *: *P* < 0.05;**; ***: *P*< 0.001.

The therapeutic efficacy of BsAb against MKN45 cell xenograft tumors was explored, and compared with PD-1 antibody and a one arm c-MET antibody. Mice were treated with IgG4, BsAb, PBMC iv + PD-1 ip antibody, or PBMC iv + BsAb ip. As shown in Figure 7A, PBMC + BsAb treatment significantly inhibited tumor size compared to treatment with BsAb alone (*P*=0.005), or PBMC + PD-1antibody (*P*=0.037). These data collectively suggest BsAb is a more potent inhibitor of tumor growth *in vivo* than PD-1 or one arm c-MET antibody.

## DISCUSSION

BsAb can redirect specific immune cells to tumor cells to enhance tumor killing, enablethe simultaneous blocking of two different antigens that exert unique or overlapping roles in pathogenesis, and can potentially increase binding specificity by interacting with two different cellular surface antigens instead of one [41]. BsAb are a promising way of enhancing anti-tumor immunity in immunotherapy with the goal of achieving synergistic effects. BsAb can interfere with multiple surface receptors or ligands associated with cancer cell proliferation or inflammatory processes. For example, BsAb simultaneously targeting EGFRx c-MET produced synergies that inhibited tumor proliferation and metastasis more effectively [42–44]. With the development of genetic engineering utilizing recombinant DNA technology, more than 30 BsAbs have so far been exploited in a clinical setting. Currently, two of the front-runners have been approved for the market by the FDA for the treatment of pro-B cell acute lymphocyte leukemia (ALL) and malignant ascites, and several more are in clinical trials [45, 46].

A novel, targeted c-MET and PD-1 humanized bispecific monoclonal antibody was developed in our laboratory, which can bind both c-MET and PD-1 cellular surface antigens with high affinity and specificity. Theoretically, our BsAb could inhibit tumor progression, migration, metastasis, and angiogenesis by blocking c-MET, and can also rescue systemic T cell function by blocking PD-1 in cancer cells overexpressing c-MET and PD-L1. Moreover, our BsAb could form a bridge between T cells and tumor cells, allowing the T cells to target the tumor cells directly.

Previous attempts to target c-MET with antibodies have met with difficulty, because most antibodies have intrinsic agonistic activity [47, 48], and the effects of individual antibodies were minimal. The divalent structure of antagonistic antibodies directed to c-MET often activates c-MET signaling pathway via receptor dimerization and cross-activation, which appear to function as agonists. Recently, however, a novel one-armed variant of the c-MET antibody 5D5 (OA5D5) was developed by Genentech (South San Francisco, CA), which acts as a pure antagonist, and can inhibit the growth of cells dependent on HGF/c-MET autocrine and paracrine signaling, and inhibit glioma growth in an orthotopic model *in vivo* [49, 50]. The monovalent format antibodies proved to be necessary against the agonistic activity [51]. In the present study, we have successfully developed a novel BsAb targeting c-MET and PD-1, using monovalent forms of anti-c-MET and anti-PD-1, which can avoid inducing c-MET receptor dimerization, and the intrinsic agonistic activity, just as OA5D5 can.

*In vitro*, our BsAb inhibited HGF-induced growth and migration of c-MET-addicted tumor cells (Figure 2A, 2B), and promoted apoptosis in tumor cells (Figure 2C), even more effectively than JNJ, a known c-MET inhibitor. BsAb also inhibited HGF-stimulated c-MET autophosphorylation of Tyr1234/1235 in the activation loop, which activates downstream molecules, such as AKT and ERK1/2 (Figure 3). It is well known that phosphorylated AKT and ERK1/2 are the two major c-MET downstream oncogenic signaling proteins [52]. PI3K/AKT signaling activation, shown to mediate essential anti-apoptosis signals, has also been reported to be critical for cell dissociation [53, 54], and p38MAPK, along with ERK1/2, promoted HGF-mediated cornea epithelial cell migration [55]. Therefore, we could conclude that BsAb inhibits the pathways of c-MET, including AKT and ERK1/2, which may contribute to the antigrowth, antimigration, and proapoptosis effects. At the same time, analysis of the effect of BsAb on c-MET protein levels suggests that BsAb can induce protein degradation, in both dose- and time-dependent manners, in cancer cells (including MKN45 and A549 cells). We also found that BsAb didn't affect the c-MET mRNA expression levels in the two cell lines (Figure 4C, 4D), which suggested that the c-MET protein degradation may result from BsAb-mediated receptor internalization in post-transcription. However, the molecular mechanisms by which pathways promote BsAb-mediated degradation of c-MET protein require further clarification in cancer cells. In sum, these data support the conclusion that BsAb neutralizes c-MET activation, not only by blocking c-MET downstream oncogenic signaling activation, but also by reducing c-MET protein levels.

Effectively activated tumor reactive T cells, and suppressed checkpoint inhibitors, have resulted in prolonged tumor regression and improved overall response rates in melanoma, renal cell cancers, and lung cancers [56]. Recently, immunotherapy by checkpoint blockade directed against the PD-1/PD-L1 pathway has shown remarkable antitumor responses in patients with advanced melanoma, lung cancer, and other cancers with durable clinical responses [23, 25, 57, 58]. T cells activated in the absence of PD-L1/PD-1 costimulation are functionally activated, and produce higher levels of Th1 cytokines, in particular IL-2, TNF-α, and IFN-γ [59]. IFN-γ could upregulate PD-L1 expression on many cell lines. In our study, we described, for the first time, a new approach to inhibit PD-1/PD-L1 costimulation by directly targeting PD-1 expression in Jurkat T cells using our BsAb. We observed that Jurkat T cells express PD-1 at low levels, but approximately 36% of Jurkat T cells expressed PD-1 after stimulation with PHA for 48 h (Figure 5A), and we have also observed that MKN45 and A549 cells retained remarkably upregulation PD-L1expression when stimulated with IFN-γ (Figure 5B). The results showed that our BsAb can significantly rescue Jurkat T cell IL-2 production, which is a key cytokine indispensable to the proliferation and survival of activated T cells. In addition, cancer cells pretreated with IFN-γ obviously inhibited IL-2 secretion by Jurkat T cells. It is well known that cancer cells might encounter IFN-γ produced by activated T cells in the tumor's local microenvironment, and thus could respond by upregulating PD-L1 on their cell membrane to suppress T cell secretion of IL-2. Our results demonstrate that BsAb can rescue PD-L1/PD-1-mediated inhibition of IL-2 in a tumor microenvironment, which may play an important role in activating effector T cells by blocking PD-L1/PD-1 signaling.

At the end of the study, we examined the effect of our novel humanized BsAb on tumor growth *in vivo* using a xenograft model. Our results demonstrated that BsAb effectively inhibited tumor growth *in vivo*, it reduced the tumor volume and weight near 60% (Figure 6A, 6B). We also found that BsAb can reduce the expression of proliferation, angiogenesis, migration, and invasion-related proteins, including Ki-67, VEGF-A, and MMP-9, which indicates its potent ability to inhibit cancer cell proliferation, angiogenesis, migration, and invasion *in vivo*. TNF-α, and IL-6 are produced in the microenvironment of the tumor as a result of the nonspecific inflammatory response, which appears to increase c-MET expression [60]. Further investigation showed that BsAb could strongly inhibit inflammatory factor production *in vivo* compared with control mice, including IL-6, although TNF-α was not reduced by BsAb, which may alleviate chronic inflammation during the progress of tumor treatment.

Our BsAbs can simultaneously bind human c-MET and PD-1 with high affinity and specificity, which should achieve synergistic effects. In this study, we found that BsAb + PBMC treatment is superior to anti-PD-1 + PBMC treatment at inhibiting tumor growth *in vivo* (Figure 7A) (*P*=0.037). We also explored the effect of c-MET antibody in the inhibition of tumor growth *in vivo*, however, it proved difficult (data not shown), which might be due to intrinsic agonistic activity. NOD/SCID mice are deficient of humanized PD-1 highly expressing T cells, so our BsAb targeting the PD-1 pathway may be useless in treating mice alone. In order to simulate c-MET antibody effects, BsAb was used alone in treating NOD/SCID mice, which may simulate a monovalent form of c-MET antibody, similar to OA5D5. The results showed that BsAb + PBMC treatment is superior to BsAb alone at inhibiting tumor growth *in vivo* (*P*=0.005) (Figure 7A, 7B). At the same time, the safety of c-MET/PD-1 BsAb in HUVEC cell viability was done by MTS assay. There were no significant difference between BsAb and IgG4 treatment (Supplementary Figure 1).

Nevertheless, our study did not directly address the effects of BsAb comparing with one-armed c-MET antibody *in vivo*. Only through comparing with the antibody, could we conclude that the BsAb is indeed superior to the one-armed c-MET antibody. Further studies are necessary to explore the hypothesized superiority of BsAb over c-MET antibody *in vitro* and *in vivo*. In addition, our observation of BsAb treatment was only limited to c-MET and PD-L1-positive tumor cells, however, we do not know whether BsAb has effects in c-MET and PD-L1-negative tumor cells. In order to obtain large numbers of humanized PD-1 highly expressing T cells, reconstituting the immune systems, human PBMC were transplanted into NOD/SCID mice by tail vein injection. However, the mice may have developed host versus graft disease (HVGD), which may influence the effect of BsAb treatment in mice.

In conclusion, BsAb treatment strongly inhibited the growth of cancer cells *in vitro* and *in vivo*, more effectively than PD-1 antibody or simulated one-armed c-MET antibody. Mechanisms responsible for this antitumor effect include inhibition of tumor cell proliferation, migration, angiogenesis, and proinflammatory cytokine secretion, as well as proapoptotic effects, which may block c-MET downstream signaling in the AKT and ERK1/2 signaling pathways, and the PD-L1/PD-1 signaling pathway. Therefore, our studies identified BsAb to be a potential antitumor agent that may be able to effectively complement immunotherapy strategies in future.

## MATERIALS AND METHODS

### Cell lines and cell culture conditions

MKN45, A549, HT-29, MDA-MB-231, 769P, and Jurkat T cells were obtained from the ATCC; IM95 cell line was obtained from JCRB Cell Bank; HN30 was a gift from the Department of Oral and Maxillofacial-Head and Neck Oncology (Shanghai Jiao Tong University School of Medicine, China); Human uveal melanoma 92.1 was a gift from the Institute of Eye Vision, Shanghai Jiao Tong University School of Medicine. HN30, 92.1, MKN45, A549, HT-29, MDA-MB-231, 769P, and IM95 were cultured in DMEM (IM95 containing 1% insulin) and Jurkat T cells cultured in RPMI- 1640 supplemented with 10% FBS (GIBCO) and of 1% penicillin/streptomycin (GIBCO). Cells were incubated at a 37°C humidified incubator containing 5% CO_2_.

### Reagents and antibodies

Human recombinant HGF, PHA, and IFN-γ were purchased from GenScript (Nanjing, China). The c-MET kinase inhibitors JNJ38877605 and rapamycin were obtained from Selleckchem (Houston, TX). Antibodies used include: c-MET mAb (#8198), phospho-MET (Tyr1234/1235) mAb (#3077), AKT mAb (#9272), phospho-AKT (Ser473) mAb (#4060), p44/42 MAP kinase mAb (#4696), phospho-p44/42 MAP kinase (Thr202/Tyr204) mAb (#4376), GAPDH mAb (#2118). Anti-mouse IgG, HRP-linked antibody (#7076), anti-human IgG4 was used as control, HRP-linked antibody (#7074) were used as secondary antibodies. All antibodies were purchased from Cell Signaling Technology, Inc. PE-labled mouse anti-human PD-1antibody was purchased from Abcam.

### RNA isolation and real-time quantitative PCR (qPCR)

Total RNA was isolated from each cell line with TriReagent (Sigma-Aldrich) according to the manufacturer's instructions. cDNA synthesis and qPCR reactions were performed with SYBR® FAST qPCR Kit Master Mix (KAPA). The primer pairs used were: c-MET, forward 5′-TTC-ACC-GCG-GAA-ACA-CCC-ATC-3′, reverse 5′-GTC-TTC-CAG-CCA-GGC-CCA-3′; PD-L1, forward 5′-TGG-CAT-TTG-CTG-AAC-GCA-TTT3′, reverse 5′-GTG-GTG-GTC-TTA-CCA-CTC-AGG3′; GAPDH, forward 5′-CAT-CTC-TGC-CCC-CTC-TGC-TGA-3′, reverse 5′-GGA-TGA-CCT-TGC-CCA-CAG-CCT-3′. ΔCT (cycle threshold) values were calculated based on the mean CT values of the target genes and mean CT values of the reference control gene GAPDH, using the following formula: ΔCT = Mean CT for Target Gene – Mean CT for GAPDH. Relative gene expression levels were calculated using ΔΔCT analysis. ΔΔCT = ΔCT of Sample – ΔCT of Calibrator. Relative Gene Expression = 2^−(ΔΔCT)^.

### Cell viability and apoptosis assay

Cells were seeded in a 96-well plate at 1×10^4^ each hole overnight and were grown in the presence of IgG4 (control) and BsAb for 8 h and JNJ for 2 h, then were treated with HGF. After 3 day, 20 μl MTS (Sango, China) was added to each sample and incubated for 4 h. The absorbance of solution was recorded at 490 nm with a thermo microplate reader. The results of the MTS assay to reflect cell viability.

Apoptotic cells were measured by flow cytometry as follows: cells were harvested and washed with PBS, resuspended in pre-diluted binding buffer, and stained with annexinV-FITC (BD Biosciences, CA, USA) for 30 min at room temperature. After being washed and resuspended in PI binding buffer, the cells were immediately subjected to apoptosis analyses by flow cytometry using Cell Quest Software.

### *In vitro* scratch wound healing migration assay

MKN45 or A549 cells were plated in a 6-well plate. After overnight a sterile 10 μl pipette tip was used to make a wound across a cell culture monolayer. Cells were incubated in DMEM-0% FBS in the presence of BsAb for 8 h and JNJ for 2 h, then were treated with HGF (100 ng/mL). Multiple photographs of the wound were taken immediately after wounding 0 h and 24 h under a phase-contrast microscopy. The efficiency of the wound healing process was determined by calculating the area of the cell gap at the indicated times (0 and 24 h), using ImageJ software. Three random images were used for each wound at each experimental point. The results are expressed as percentage of healing at 24 h with respect to zero time.

### Western blotting

To determine the molecular mechanism of HGF on c-MET signaling pathway, Western blot analysis was performed to detect key proteins involved the pathway. The cells were lysed with M-PER® Mammalian Protein Extraction (Pierce). Proteins were quantified by the BCA Protein Assay kit (Pierce) according to the manufacturer's instructions. Samples containing a total of 50 μg protein were incubated at 100°C for 5 min, separated by SDS-polyacrylamide gel electrophoresis, and subsequently electrotransferred onto a polyvinylidene difluoride membrane. Essential component detection in the cells was performed with antibody at overnight incubation at 4°C, and then HRP-conjugated secondary antibody (1:5,000 dilution; Pierce Chemical) was added for 1 h at room temperature, followed by the development of reactions in a chemiluminescent detection system. GAPDH antibody was used as the control.

### Human PBMC preparation and transplantation

Blood from healthy volunteers was collected in heparinized tubes. For the isolation of PBMCs, blood was diluted 1:1 with RPMI1640 medium (vol/vol) prior to transferring into the leucosep tube. Following centrifugation (20 min, 2000 rpm), the PBMC layer was pooled and transferred into a 15 ml falcon tube. The sample was washed with 10 ml phosphate-buffered saline (PBS) and centrifuged again for 10 min at 1500 rpm. The obtained cell pellet was resuspended in PBS. A total of 1×10^8^ PBMCs per mouse were injected into NOD/SCID mice through tail vein for the reconstitution of immune system.

### Tumor xenograft study

Approximately 5- to 6-week male NOD/SCID mice (about 20 g) were obtained from SLAC Laboratory Animal Co., Ltd. (Shanghai, China) and were kept in a specific pathogen-free (SPF) facility. They are absent of T, B lymphocytes, and NK cells. All animal treatment were in accordance with international ethics guidelines and the National Institutes of Health Care and Use of Laboratory Animals. This study was approved by the Institutional Animal Care and Use Committee of Fudan University. MKN45 cells (5×10^6^cells) were subcutaneously injected into the right flank region of mice. After 7 days, allowing the tumors to grow to about 50 mm^3^, mice were randomized into control and treatment groups. For MKN45 xenograft, mice were treated as follows: BsAb (10 mg/kg, twice per week), IgG4 and anti-PD-1 (5 mg/kg, twice per week); PBMCs (1×10^8^) were injected the first and fourth time drugs were administered through via tail vein. Each group consisted of 5-6 mice. At the same time, body weight and tumor size were measured using an electronic balance and a vernier caliper. Tumor volume was calculated using the formula: volume = (length × width^2)^)×0.5. After the endpoint (28 days after the implantation), blood was collected from the aorta abdominalis and the serum was separated and frozen at −80°C for further analyses. Mice were then sacrificed for the collection of tumors.

### Immunohistochemistry (IHC)

Tumor samples were fixed in 4% formalin, embedded in paraffin, and sectioned into 4-μm-thickness slices. After dewaxing, tissues sections were processed by antigen retrieval, followed by the quenching of endogenous peroxidase activity using hydrogen peroxide. PBS (0.1% Tween 20) containing anti-goat serum was used to block non-specific binding sites. Slides were incubated with polyclonal antibodies against Ki-67 or VEGF-A or MMP-9 (in 1:100 dilution, CST, US). After washing with PBS, slides were incubated with biotinylated anti-rabbit IgG antibody followed by horseradish peroxidase-conjugated streptavidin. After developing in DAB substrates (Invitrogen, US), 5 high-magnification fields were randomly selected in each slide, and the numbers of positive cells in each field were counted (each group=4) by the image analysis system (Image-Pro Plus).

### ELISA

The cytokines levels of IL-2 (culture supernatants), TNF-a, and IL-6 (mice serum) were measured using the specific ELISA kits (R&D Systems) following the manufacturer's instructions.

### Statistical analysis

The statistical analysis between groups was performed using GraphPad Prism 5 software. The unpaired 2-tailed *t* test was used for the comparison and the level of significance was defined when *P* < 0.05.

## SUPPLEMENTARY MATERIALS FIGURE


