# Supplementary material for: A novel bispecific c-MET/PD-1 antibody with therapeutic potential in solid cancer
Source: Oncotarget. 2017 Mar 14;8(17):29067–79. doi: 10.18632/oncotarget.16173 (PMC5438713; doi:10.18632/oncotarget.16173)
Supplement: Supplementary file 1 [file oncotarget-08-29067-s001.pdf]

A novel bispecific c-MET/PD-1 antibody with therapeutic potential in solid cancer

SUPPLEMENTARY MATERIALS

SUPPLEMENTARY FIGURE

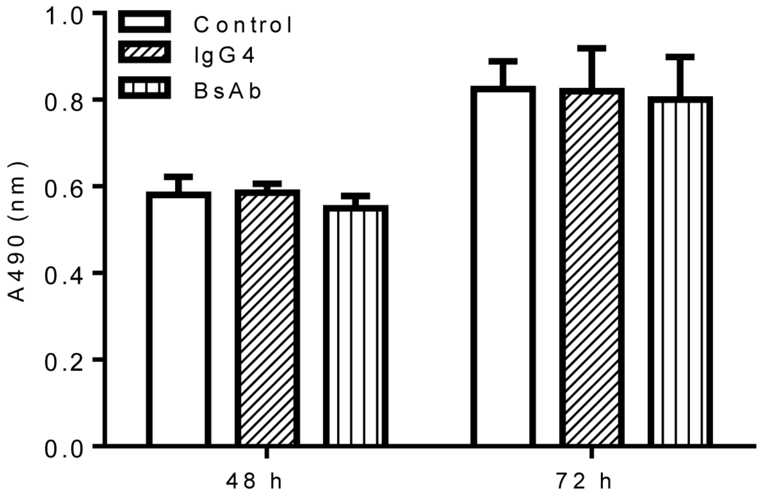

Supplementary Figure 1: Safety evaluation of BsAb on HUVEC cells. HUVEC cells were seeded in a 96-well plate at 1×10^4 each hole overnight and were grown in the presence of PBS (control), IgG4 (0.5 μM) or BsAb (0.5 μM). The viability of HUVEC cells were assessed by MTS assay after treatment with BsAb for 48 h and 72 h. MTS (20 μl) was added to each sample and incubated for 4 h. The absorbance of solution was recorded at 490 nm with a thermo microplate reader. The results of the MTS assay to reflect cell viability. Each experiment was repeated 3 times and the results were shown mean ± SD.
